# High-Density Lipoproteins and the Kidney

**DOI:** 10.3390/cells10040764

**Published:** 2021-03-31

**Authors:** Arianna Strazzella, Alice Ossoli, Laura Calabresi

**Affiliations:** Centro E. Grossi Paoletti, Dipartimento di Scienze Farmacologiche e Biomolecolari, Università degli Studi di Milano, 20133 Milano, Italy; arianna.strazzella@unimi.it (A.S.); alice.ossoli@unimi.it (A.O.)

**Keywords:** chronic kidney disease, high-density lipoprotein, lecithin–cholesterol acyltransferase

## Abstract

Dyslipidemia is a typical trait of patients with chronic kidney disease (CKD) and it is typically characterized by reduced high-density lipoprotein (HDL)-cholesterol(c) levels. The low HDL-c concentration is the only lipid alteration associated with the progression of renal disease in mild-to-moderate CKD patients. Plasma HDL levels are not only reduced but also characterized by alterations in composition and structure, which are responsible for the loss of atheroprotective functions, like the ability to promote cholesterol efflux from peripheral cells and antioxidant and anti-inflammatory proprieties. The interconnection between HDL and renal function is confirmed by the fact that genetic HDL defects can lead to kidney disease; in fact, mutations in apoA-I, apoE, apoL, and lecithin–cholesterol acyltransferase (LCAT) are associated with the development of renal damage. Genetic LCAT deficiency is the most emblematic case and represents a unique tool to evaluate the impact of alterations in the HDL system on the progression of renal disease. Lipid abnormalities detected in LCAT-deficient carriers mirror the ones observed in CKD patients, which indeed present an acquired LCAT deficiency. In this context, circulating LCAT levels predict CKD progression in individuals at early stages of renal dysfunction and in the general population. This review summarizes the main alterations of HDL in CKD, focusing on the latest update of acquired and genetic LCAT defects associated with the progression of renal disease.

## 1. Introduction

High-density lipoproteins (HDL) are a highly heterogeneous class of lipoproteins, with subclasses differing in density, size, shape, and composition. HDL heterogeneity is the result of the action of several plasma and cellular factors such as enzymes, transfer proteins, and membrane receptors and transporters [1]. Among these factors, lecithin–cholesterol acyltransferase (LCAT) plays a central role, being the only enzyme able to esterify cholesterol in plasma lipoproteins, mainly in HDL [2]. Kidney contributes to HDL catabolism, being the major site of apoA-I and small HDL particles degradation. These particles can be removed from the circulation by glomerular filtration, as their size allows the crossing of the glomerular barrier [3]. In the proximal tubule, apoA-I is entirely reabsorbed by the action of cubilin, a multiligand receptor expressed in the apical membrane of kidney tissue which binds apoA-I with high affinity and mediates its endocytosis [4]. There is evidence that renal cubilin-mediated uptake of apoA-I from the glomerular filtrate might be part of a salvage process that affects plasma apoA-I/HDL levels [5].

The link between plasma HDL and the kidney is bidirectional. On one side, chronic kidney disease (CKD) affects plasma HDL levels, HDL structure and subclass distribution, and HDL functionality; on the other side, inherited HDL disorders can lead to kidney dysfunction, as in LCAT deficiency.

This review addresses the current knowledge on the two aspects: (i) how CKD affects HDL structure, composition, and functionality, and (ii) how HDL defects affect renal function.

## 2. HDL in CKD

### 2.1. Dyslipidemia in CKD

CKD is a pathological condition characterized by the presence of kidney damage or reduction of the glomerular filtration rate (GFR) for more than 3 months and it is classified into five stages based on the values of GFR [6]. In 2017, the cases of all-stage CKD recorded reached the global prevalence of 9.1% [7]. The onset of CKD can be attributable to a wide range of different risk factors, including diabetes, hypertension, oxidative stress, and inflammation. Cardiovascular disease (CVD) represents the first cause of mortality in CKD patients and is related to a wide range of risk factors, including diabetes, hypertension, inflammation, and oxidative stress [8]. The risk of cardiovascular events gets higher as the kidney function declines [9]. Dyslipidemia is a major CVD risk factor in the general population and is frequently observed among patients with CKD. The alterations in lipid profile of CKD patients include increased triglycerides and decreased HDL-cholesterol (HDL-c) levels, while the levels of low-density lipoprotein (LDL)-cholesterol are within or below the normal range [10]. Hypertriglyceridemia is one of the most common quantitative lipid abnormalities and starts at early stages of CKD. Indeed, some clinical studies have reported that plasma triglycerides concentration increases in patients with impaired renal function when serum creatinine levels and glomerular filtration rate are still in the normal range [11,12]. Reduced catabolism of triglyceride-rich lipoproteins (very-low-density lipoproteins (VLDL), chylomicrons, and their remnants), as observed in individuals with predialysis CKD [13], and increased liver secretion of these particles [14] have been suggested to explain the hypertriglyceridemia in kidney disease.

Reduced HDL-c concentration is a typical trait of the CKD dyslipidemia. The reduction of HDL-c and apoA-I concentrations observed in patients with CKD are partly explained by the downregulation of apoA-I synthesis by the liver [15] but also by the defective LCAT concentration and activity, leading to altered plasma HDL remodeling [16]. The maturation of HDL particles is impaired in CKD due to a severely delayed LCAT-dependent conversion of nascent discoidal preβ-HDL into mature spherical α-HDL [16,17], resulting in the accumulation of preβ-HDL in the plasma of CKD patients [18,19]. Other factors contribute to increase lipid-poor HDL particles in the plasma of CKD patients, like their impaired renal clearance [20,21] and the elevated concentration of triglyceride-rich lipoproteins [22], which represent a source of nascent HDL.

Plasma HDL in patients with CKD show a selective reduction of LpA-I–A-II particles [16], likely explained by an accelerated catabolism of these particles when LCAT activity is reduced, as observed in genetic LCAT deficiency [23].

The proteome composition of HDL in CKD is altered, as recently reviewed by Marsche et al. [20]. Specific HDL proteins, such as serum amyloid 1 (SSA1) and apoC-III, are increased in CKD patients [24,25,26]. SSA1 is one of the major acute-phase proteins secreted during inflammation and is an atherogenic mediator [27]; apoC-III is an inhibitor of lipoprotein lipase and a strong predictor of CVD risk [28,29]. Moreover, HDL from patients on hemodialysis present an increased amount of albumin, lipoprotein-associated phospholipase A2, apoA-IV, α-1-antitrypsin, retinol-binding protein 4 and α-2 catenin, and lysophospholipids [30]. Besides the enrichment in these components, a reduction in apoA-I, apoA-II, apoC-I, apoM, and paroxonase-1 (PON 1) has been reported consistently [30,31,32,33].

The structural modifications observed in HDL are associated with the impairment of fundamental HDL functions. HDL capacity to promote cholesterol efflux from macrophages, a parameter inversely associated with the likelihood of coronary artery disease independently of HDL-c levels [34], is impaired in CKD patients [33,35], likely due to the HDL enrichment in SSA1 and apoC-III and the depletion in apoA-I, apoA-II, and phospholipids [20,36,37]. HDL ability to maintain correct endothelial homeostasis is also compromised in CKD patients because of chronic inflammation and oxidative stress; moreover, Speer et al. have demonstrated that in CKD patients, HDL are not only dysfunctional but become “toxic” for the accumulation of asymmetric dimethylarginine which enhances reactive oxygen species (ROS) and suppresses endothelial NO bioavailability by activating the Toll-like receptor-2 pathway [38].

**Figure 1:**
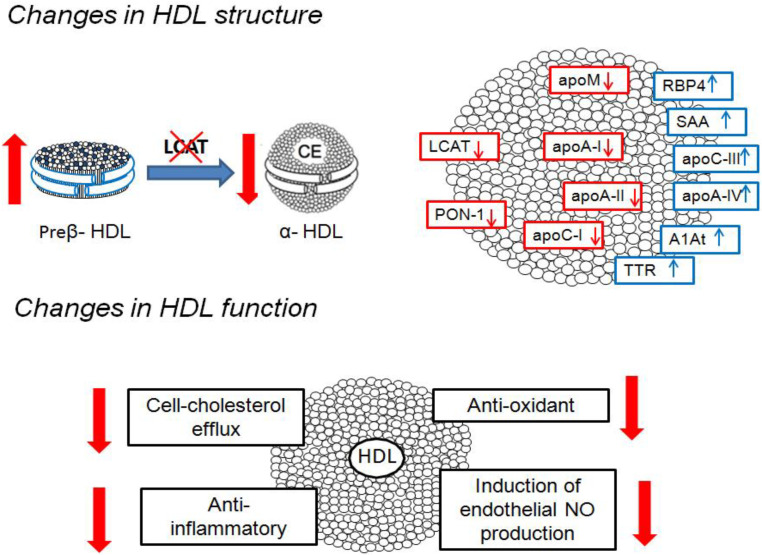
High-density lipoprotein (HDL) structural and functional alterations in chronic kidney disease (CKD). Upper panel: major modifications in HDL shape and protein composition; lower panel: altered HDL functions. Abbreviations: LCAT, lecithin–cholesterol acyltransferase; PON-1, paroxonase-1; apoA-I, apolipoprotein A-I; apoA-II, apolipoprotein A-II; apoC-I, apolipoprotein C-I; apoM, apolipoprotein M; apoC-III, apolipoprotein C-III; apoA-IV, apolipoprotein A-IV; SAA, serum amyloid A; A1AT, α-1-antytrypsin; RBP4, retinol-binding protein 4; TTR, transthyretin. The arrows indicate increase or decrease.

HDL antioxidant ability is also impaired in patients with CKD [33,39], likely due to a reduction in PON-1 and apoA-I content, two molecules known to exert antioxidant activity [40], and a concomitant enrichment of acrolein-modified apoA-I [41]; this latter modification also contributes to explain the reduced HDL-mediated cholesterol efflux [31,42]. Finally, in patients with CKD, a loss of the anti-inflammatory activity of HDL has been reported, explained at least in part by the enrichment in acute-phase proteins, such as SAA [43], and in apoC-III, involved in the activation of inflammatory cell response and organ damage by alternative inflammasome activation [44] (Figure 1).

### 2.2. HDL in CKD Progression

The described quantitative and qualitative alterations of the HDL system worsen with the progression of CKD [45]. Importantly, the reduction of HDL-c is the only lipid abnormality associated with the progression of renal disease in mild-to-moderate CKD patients independently of classical risk factors such as diabetes and hypertension [46]. The association between low HDL-c concentration and progression of CKD has been demonstrated in hemodialysis patients, who present a further significant reduction in HDL-c, apoA-I, and apoA-II concentrations compared with CKD patients at a lower stage of the disease [16]. A large epidemiological study, involving a cohort of two million American veterans with a median follow-up of 9 years, showed that individuals with plasma HDL-c levels <30 mg/dL have a 10–20% higher risk for incident CKD and/or CKD progression compared with individuals with HDL-c ≥40 mg/dL [47]. Furthermore, the data collected highlight the existence of a U-shaped relationship between HDL-c levels and incidence of CKD or CKD progression in the general population, in which the risk of adverse renal outcome is increased not only in the lowest deciles of HDL-c concentration but also in the highest ones [47]. These findings have been confirmed by several observational studies showing a paradoxical association between elevated HDL-c levels and cardiovascular events or mortality in patients undergoing hemodialysis [48,49]. The association between HDL-c level and CKD progression in non-dialysis patients with CKD is instead conflicting; the Chronic Renal Insufficiency Cohort (CRIC) study showed that the HDL-c level was not independently associated with the composite end point of end-stage renal disease (ESRD) or a 50% reduction in estimated (e)GFR in 3939 adults with CKD [50]; moreover, the causality or consequentiality of the association between HDL-c and CKD outcomes still needs to be confirmed [24].

### 2.3. LCAT in CKD

Animal studies have firstly shown that chronic kidney failure is associated with hepatic downregulation of the *LCAT* gene, which leads to an impaired LCAT activity [51]. Moreover, a study on rats with nephrotic syndrome highlighted a reduction in plasma LCAT activity due to urinary excretion of the enzyme, leading to a condition of acquired LCAT deficiency [51]. A reduction in LCAT concentration/activity has later been demonstrated in CKD patients at different stages of the disease [16], confirming that CKD is associated with an acquired LCAT deficiency in humans. Reduction in LCAT leads to a defective cholesterol esterification, impaired preβ-HDL maturation, and accelerated catabolism of LpA-I–A-II particles, as shown in individuals with genetic LCAT deficiency [52]. Since the alterations of the lipid/lipoprotein profile in LCAT deficiency are involved in the pathogenesis of renal disease [53], a recent study by Baragetti et al. investigated whether the reduced LCAT concentration in CKD patients could affect the progression of renal damage [54]. The results demonstrated that reduced circulating LCAT levels predict CKD progression in individuals at early stages of renal dysfunction independently of changes in HDL-c levels, confirming that changes in HDL subclass distribution contribute to the progression of renal damage [54]. Interestingly, the same study demonstrated that baseline low LCAT concentration predicts kidney function impairment, measured as dialysis entry and/or basal creatinine level doubling, also in the general population [54]. Individuals with low LCAT concentration at the baseline had decreased HDL-c and high preβ-HDL, and their sera showed a damaging effect on podocytes and tubular cells [54]. The same study also showed that in vitro incubation of low-LCAT subjects’ sera with recombinant human LCAT can correct HDL abnormalities and reduce the damaging effects in renal cells [54], thus confirming the causal relation between low LCAT concentration and toxic effects.

## 3. Genetic HDL Defects and CKD

Glomerular lipidosis is a condition characterized by a lipid deposition which can be mediated by infiltrating macrophages or directly affected by resident glomerular cells [55]. This renal alteration can be observed in various diseases of non-genetic origin, such as diabetic glomerulosclerosis [56], but it can also occur in genetic diseases, like LCAT deficiency [53]. This observation strongly supports the existence of a relation between lipid alterations and kidney disease development and progression, even if the nature of this link is still not completely understood.

Mutations in genes codifying for key proteins in lipid metabolism and HDL function, like apoA-I, apoE and apoL, have been associated with alterations in renal function (Table 1).

ApoA-I is the major protein component of HDL in plasma. Several mutations in the *APOA1* gene have been identified: some of them are associated with reduced LCAT activity, leading to a strong reduction of HDL levels and a high risk of early-onset cardiovascular disease, while other mutations exert an amyloidogenic effect, increasing the propensity of the encoded protein to form insoluble fibrils, which can deposit in different organs including the kidney, leading to mild renal dysfunction which can progress to end-stage kidney failure [57,58,59].

ApoE is a structural key regulatory protein of chylomicrons, LDL, VLDL, and HDL [60]. Mutations in the *APOE* gene have been associated with lipoprotein glomerulopathy, a rare form of renal lipidosis that leads to nephrotic syndrome [61]; histologically, this condition is characterized by abnormal lipoprotein deposition in glomerular capillaries and mesangial proliferation [62]. Moreover, the relative allele frequency of apoE affects renal functionality in different ways: for example, Oda et al. have demonstrated that apoE2 has higher frequency in patients with ESRD, to the disadvantage of a reduced frequency of apoE4 [63]. Subsequent studies have confirmed that the ε2 allele of the *APOE* gene might increase the risk of ESRD [64]. Finally, a reduction of *APOE* expression at the glomerular level has been described in focal and segmental glomerulosclerosis and may contribute to the pathogenesis of the disease, without a pathogenetic role of genetic variants [65]. All these results suggest that alterations in the *APOE* gene could be involved in the increased susceptibility to glomerular damage, but the eventual cause–effect relationship needs further investigations.

ApoL1 is an apolipoprotein, encoded by the *APOL1* gene, which co-localizes with apoA-I in HDL particles [66]. Two variants of the *APOL1* gene, named G1 and G2, have been associated with an increased risk of developing renal disease in individuals of African ancestry [67,68]. Genotypes presenting the two variant alleles in any combination (homozygous G1/G1, homozygous G2/G2, or compound heterozygous G1/G2) are associated with higher risk of renal disease, while heterozygous subjects have no or only minimally increased risk [55,69]. *APOL1* risk variants present a high frequency in subjects of African ethnicity affected by focal segmental glomerulosclerosis, HIV-associated nephropathy, and nondiabetic end-stage kidney disease; moreover, carriers of high-risk genotypes show a strongly increased odds ratio for these diseases compared to individuals carrying *APOL1* low-risk genotypes [68,69,70]. Several pathogenic mechanisms have been proposed to explain the relationship between *APOL1* risk variants and renal toxicity. Studies on renal cells or animal models have demonstrated that the expression of *APOL1* G1 and G2 variants is associated with a number of processes contributing to the increase of renal damage, such as mitochondrial dysfunction and reduced maximum respiration rate [71,72], induction of the stress-activated protein kinases p38 MAPK and JNK [73], activation of protein kinase R pathway [74], and induction of podocytes injury via activation of autophagy [75,76].

### 3.1. Genetic LCAT Deficiency

Familial LCAT deficiency (FLD, OMIM# 245900) is a rare autosomal recessive condition caused by loss-of-function mutations in the gene encoding LCAT. FLD patients are characterized by markedly low plasma HDL-c levels. Clinical manifestations of the disease include corneal opacity, anemia, and proteinuria, which usually progresses to renal failure by the fourth decade of life. Loss-of-function mutations in the *LCAT* gene are also the cause of fish-eye disease (FED, OMIM# 136120). Similar to FLD, FED is also characterized by low HDL-c levels; however, FED patients are generally spared from renal disease and experience relatively more benign outcomes [53]. More than one hundred mutations in the *LCAT* gene have been identified to date, spread all along the *LCAT* gene [77]. Mutations in the *LCAT* gene leading to the lack of protein production or to the production of a completely inactive LCAT enzyme cause FLD [53,77]. Mutations characterized by the production of an enzyme lacking activity on the HDL substrate (α-LCAT activity) but retaining activity on apoB-containing lipoproteins (β-LCAT activity) cause FED [53,77]. The differential diagnosis of FLD and FED is limited to carriers of two mutant *LCAT* alleles and requires the distinct in vitro measurement of α-LCAT activity using a standardized exogenous HDL and the cholesterol esterification rate in plasma lipoproteins that include both α and β-LCAT activity [52]. Both measurements are null in FLD cases, whereas in FED cases, only α-LCAT activity is null [52]. Carriers of one mutant *LCAT* allele cannot be classified as FLD or FED based on biochemical criteria, and differential diagnosis requires the expression of LCAT mutants in cultured cells and subsequent measurement of LCAT activities in cell media [78].

From a biochemical point of view, FLD patients present abnormal plasma lipid and lipoprotein profiles characterized by an increased amount of unesterified cholesterol, low levels of HDL-c, a preponderance of small discoidal pre-β HDL particles, and the presence of LpX, an abnormal lipoprotein principally composed of phospholipids (60%) and unesterified cholesterol (30%) [79]. Under electron microscopy analysis, LpX appears as a multilamellar vesicle with bi- or multi-layer phospholipids. The size of LpX is variable, and its diameter ranges from 30 to 70 nm [79]. Unfortunately, there is no routine clinical laboratory method for detecting LpX, whose presence can be detected by agarose gel, but cannot be quantified.

Renal disease, which ultimately progresses to end-stage renal disease, is the major cause of morbidity and mortality in FLD patients. Early in the disease course, FLD patients develop proteinuria and focal segmental glomerulosclerosis. Kidney biopsies from FLD patients are characteristic of the disease, with the presence of mesangial expansion, a mild increase in mesangial cellularity, and irregular thickening of the glomerular capillary walls [80]. Lipid deposits of unesterified cholesterol and phospholipids with resulting vacuolization of the glomerular basement membrane and a typical ‘foamy’ appearance are also detected [80]. These lipid deposits could contribute to accelerating the injury by activating complement proteins; indeed, immunofluorescence microscopy analysis highlights the presence of IgM deposit and bright granular staining for C3 in the capillary loops, mesangium, and arteriolar walls [81,82]. Electron microscopy analysis highlights also modification in podocytes structure with fused endothelial foot processes [83].

The pathogenesis of the renal disease in FLD is not completely understood. Animal models of the disease are complicated by the lack of kidney injury in *Lcat^−/−^* mice, the mouse model in which the expression of the *LCAT* gene is downregulated (LCAT knockout mouse). It is important to highlight that *Lcat^−/−^* mice do not spontaneously produce LpX [84]. Trying to induce LpX and renal disease, high-fat, high-cholesterol diets have been administered to *Lcat^−/−^* mice, and kidney lesions were only detected in the group of mice that accumulated LpX [84]. A different animal model has been obtained by crossing *Lcat^−/−^* mice with sterol regulatory element binding protein (SREBP) 1a transgenic mice (S+) [85]. These mice present a dramatic increase in hepatic lipogenesis and overproduction of VLDL resulting in plasma LpX. When kept on regular chow diet, LCAT-KO/SREBP1a transgenic mice spontaneously developed renal abnormalities similar to those seen in FLD patients [85]. The direct causal role of LpX in renal disease development has been demonstrated by a study showing that injection of LpX in *Lcat^−/−^* mice for 4 weeks led to proteinuria and typical kidney histological hallmarks [86].

In humans, LpX appears to become trapped in capillary loops of the glomerulus and to induce endothelial damage and vascular injury [82]. An anecdotic case reported a significant amelioration of renal function in a FLD patient during pregnancy associated with the disappearance of LpX in the plasma [87]. Finally, the absence of renal disease in FED is presumably prevented by the residual LCAT activity and the lack of formation of a significant amount of LpX [52].

Table 1 summarizes the impact of mutations in HDL-related genes on renal outcome.

### 3.2. CKD Progression in LCAT Deficiency

The rate of progression of renal disease in FLD patients is unpredictable, with some patients quickly going from mild proteinuria to a rapid deterioration in renal function even in the second decade of life [88]. Proteinuria can develop early in life, and during the third and fourth decade of life, FLD patients can develop renal failure with symptomatic edema and hypertension. Indices of renal function such as serum creatinine and creatinine clearance usually remain normal in the first three decades of life [88]. Due to the rarity of the disease, the natural history of FLD is still largely unknown; however, a recent work has analyzed the progression of the disease in 18 FLD carriers followed, in some cases, for more than two decades [89]. In this Italian cohort, half of the FLD patients had the first renal event, classified as kidney failure or kidney transplantation or death for renal complications, by the age of 46 years, with median time to a second event of 10 years [89]. Even within the same family, the development and progression of renal damage in FLD patients are highly variable [52,77], suggesting that different genetic and environmental factors can impact on kidney deterioration. Interestingly, in this cohort the plasma level of unesterified cholesterol at diagnosis predicted the rate of progression of renal events (dialysis, kidney transplantation, or death for renal complications), and patients with unesterified cholesterol values above the median showed lower event-free survival [89]. The excess of unesterified cholesterol in these patients accumulates in LpX, which deposits in the kidney [86].

No established therapy is currently available for FLD patients, and pharmacological approaches are aimed at correcting the dyslipidemia typically associated with the disease and at delaying the evolution of chronic nephropathy. Renal transplantation represents an option in FLD cases with kidney failure; however, because transplantation does not correct the underlying enzymatic defect, the disease can rapidly reoccur [89]. Enzyme replacement therapy represents an option, and recombinant LCAT is currently under clinical development [90,91]. Small-molecule LCAT activators, eventually orally active, have been tested in vitro and proved to be able to activate not only wild-type but also some mutant LCAT [92,93], but their development is still in the preclinical phase. Finally, the HDL mimetic CER-001 recently proved to reduce albuminuria and increase podocyte functionality in a mouse model of FLD [94] and to stabilize renal function in an FLD patient [95].

## 4. Conclusions

In the last decades, a clear connection between the HDL system and the kidney has clearly emerged. The kidney plays a role in the catabolism of HDL, which are cyclically interconverted in plasma by the action of a number of factors, with formation of delipidated apoA-I and small HDL that can be filtered by the glomerulus and reabsorbed by the cubilin receptor. Chronic kidney dysfunction is associated with reduced HDL-c levels and significant alterations in HDL structure and function, which worsen with the progression of the renal disease. The levels of the LCAT enzyme, a major player in HDL metabolism, are also reduced in CKD, and its plasma concentration is associated with disease progression. This is not surprising, since genetically reduced LCAT levels and activity, as in familial LCAT deficiency, lead to severe HDL defects and, importantly, to renal dysfunction.

In conclusion, given the role of low HDL levels in the progression of CKD and the predictive role of LCAT concentration in CKD onset or progression, therapeutic strategies aimed at activating LCAT could restore HDL levels and function, potentially slowing CKD progression.

## Figures and Tables

**Table 1:** Mutations in genes codifying for key components of HDL lead to defective renal function.

Gene	Mutations	Impact on Renal Outcome	References
*APOA1*	Amyloidogenic mutations	Carriers develop systemic and renal amyloidosis. Renal disease starts as mild renal dysfunction, which in a percentage of subjects can reach end-stage kidney failure. Histologically, renal damage is primarily characterized by tubulointerstitial nephritis.	[58,59]
*APOE*	LPG-associated mutations	Carriers present LPG, a rare form of renal lipidosis that leads to nephrotic syndrome, usually present proteinuria and hypertension, with impairment of renal function. Glomeruli present an abnormal lipoprotein deposition in glomerular capillaries and mesangial proliferation.	[61,62]
*APOL*	G1 and G2 high-risk variants	Homozygous carriers of high-risk genotypes have an increased odds ratio for developing FSGS, HIVAN, and non-diabetic end-stage kidney disease. Moreover, overexpression of the risk variants is reported in subjects affected by these pathologies.	[68,69,70]
*LCAT*	FLD mutations	Homozygous FLD carriers develop renal disease, characterized by proteinuria and FSGS until the stage of kidney failure.	[80,81,82]

Main renal dysfunctions associated with genetic mutations in genes codifying for ApoA-I, ApoE, ApoL, and LCAT, fundamental components of HDL; Abbreviations: LPG, lipoprotein glomerulopathy; FSGS, focal segmental glomerulosclerosis; HIVAN, HIV-associated nephropathy; FLD, familial LCAT deficiency.
